# The Role of Antioxidants in the Therapy of Cardiovascular Diseases—A Literature Review

**DOI:** 10.3390/nu16162587

**Published:** 2024-08-06

**Authors:** Ewelina Młynarska, Joanna Hajdys, Witold Czarnik, Piotr Fularski, Klaudia Leszto, Gabriela Majchrowicz, Wiktoria Lisińska, Jacek Rysz, Beata Franczyk

**Affiliations:** 1 Department of Nephrocardiology, Medical University of Lodz, Ul. Zeromskiego 113, 90-549 Lodz, Poland; joanna.hajdys@gmail.com (J.H.); witold.czarnik@stud.umed.lodz.pl (W.C.); piotr.fularski18@wp.pl (P.F.); claudial@op.pl (K.L.); gabrysia.majchrowicz@gmail.com (G.M.); lisinskawiktoria324@gmail.com (W.L.);; 2 Department of Nephrology, Hypertension and Family Medicine, Medical University of Lodz, Ul. Zeromskiego 113, 90-549 Lodz, Poland; jacek.rysz@umed.lodz.pl

**Keywords:** antioxidants, oxidative stress, reactive oxygen species, cardiovascular diseases, antioxidative therapy

## Abstract

Antioxidants are endogenous and exogenous substances with the ability to inhibit oxidation processes by interacting with reactive oxygen species (ROS). ROS, in turn, are small, highly reactive substances capable of oxidizing a wide range of molecules in the human body, including nucleic acids, proteins, lipids, carbohydrates, and even small inorganic compounds. The overproduction of ROS leads to oxidative stress, which constitutes a significant factor contributing to the development of disease, not only markedly diminishing the quality of life but also representing the most common cause of death in developed countries, namely, cardiovascular disease (CVD). The aim of this review is to demonstrate the effect of selected antioxidants, such as coenzyme Q10 (CoQ10), flavonoids, carotenoids, and resveratrol, as well as to introduce new antioxidant therapies utilizing miRNA and nanoparticles, in reducing the incidence and progression of CVD. In addition, new antioxidant therapies in the context of the aforementioned diseases will be considered. This review emphasizes the pleiotropic effects and benefits stemming from the presence of the mentioned substances in the organism, leading to an overall reduction in cardiovascular risk, including coronary heart disease, dyslipidaemia, hypertension, atherosclerosis, and myocardial hypertrophy.

## 1. Introduction

**Figure 1:**
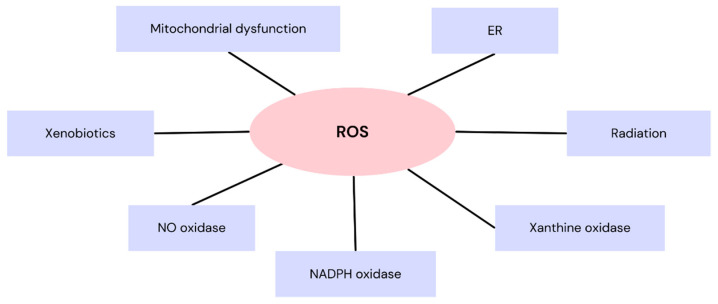
Selected sources of reactive oxygen species [11]. Abbreviations: ROS—reactive oxygen species; NADPH—nicotinamide adenine dinucleotide phosphate; NO—nitric oxide; ER—endoplasmic reticulum.

Reactive oxygen species (ROS) are small, reactive agents, generated both under pathological and physiological processes. ROS function as a secondary signaler and take part in modulating certain biological functions, including regulation of cell death. This is particularly significant because disruption of cell death regulation contributes to the development of the leading cause of death globally, namely, cardiovascular disease (CVD), and this process is disturbed as a result of the occurrence of oxidative stress, secondary to overproduction of ROS [1,2,3]. ROS vigorously engage with numerous molecules, encompassing various small inorganic compounds, carbohydrates, lipids, proteins, and nucleic acids. This may result in irreversible degradation of the target molecule’s function [4]. Apart from regulating cell death, ROS also participate in modulating the inflammatory response, vascular tone regulation, oxidation of LDL-cholesterol (LDL-c), and cell growth. Their concentration in the arterial wall increases in conditions such as diabetes, dyslipidemia, arterial hypertension, and cigarette smoking, contributing to the development of atherosclerosis [5]. Thereby, they may contribute to the onset of metabolic syndrome [6]. The role of ROS in the development and progression of atherosclerosis also encompasses DNA oxidation in vessel wall cells, endothelial dysfunction, and exertion of a negative impact on fibrous cap stability. This provokes atherosclerotic plaque ruptures and may adversely affect the frequency of cardiovascular complications, such as myocardial infarction [7]. ROS can also cause a decrease in the bioavailability of nitric oxide, thereby reducing endothelium-dependent vasodilatation, contributing to the development of arterial hypertension [8]. They also exhibit a negative impact on ryanodine receptor type 2 (RyR2), responsible for regulating calcium ion homeostasis in the atria of the heart, inducing its dysfunction, which may further contribute to the development of atrial fibrillation (AF) [9]. Other disturbances can also be observed in cardiac tissue, where ROS initiate signaling cascades implicated in inflammation, impaired contractility, interstitial fibrosis, or myocardial hypertrophy, influencing cellular architecture and function, and playing a role in cardiac injury. All of the aforementioned mechanisms contribute to the development of CVD [10]. Sources of ROS include mitochondrial dysfunction, nicotinamide adenine dinucleotide phosphate (NADPH) oxidase, xanthine oxidase, nitric oxide oxidase, endoplasmic reticulum (ER), or others, such as xenobiotics, radiation, polluted air, chemicals, or certain medications [11]. Selected sources of ROS are presented in Figure 1 [11].

Currently, CVD is a major cause of both low quality of life and mortality in developed countries, with oxidative stress playing an undisputed role in this regard [12]. However, the organism is not defenseless against ROS. It possesses a range of antioxidants, which are substances that slow down or completely inhibit the oxidation process, even when their concentration is relatively low. Antioxidants can be categorized into endogenous and exogenous types, as well as those that indirectly eliminate ROS by modulating various signaling pathways and those that directly scavenge ROS. Endogenous ones are also subdivided as enzymatic and non-enzymatic [13]. Within the category of endogenous enzymatic antioxidants, the main representatives include superoxide dismutase (SOD), which is present in every cell of the body, followed by glutathione peroxidase (GPx) and catalase (CAT). Non-enzymatic antioxidants include, among others, vitamin E (alpha-tocopherol), vitamin A (beta-carotene), vitamin C (ascorbic acid), uric acid, glutathione (GSH), flavonoids, albumin, and ceruloplasmin [14]. In addition to the body’s natural antioxidative capacities, there is increasing interest in the potential utilization of antioxidants in the therapy of diseases rooted in oxidative stress, including CVD [15]. The aim of this review is to demonstrate the effect of selected antioxidants, such as coenzyme Q10 (CoQ10), flavonoids, carotenoids, or resveratrol, in reducing the incidence and progression of CVD. In addition, new antioxidant therapies in the context of the aforementioned diseases will be considered. This review did not use specific selection criteria or research methodology to identify sources and references.

## 2. Coenzyme Q10

### 2.1. Chemical Characteristics

**Figure 2:**
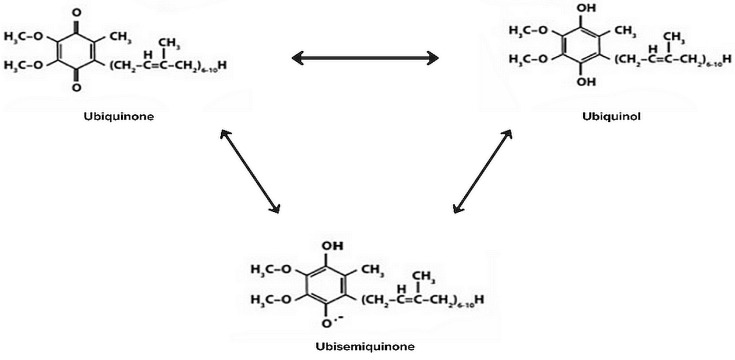
Existing forms of CoQ10.

CoQ10 is a naturally occurring lipid-soluble quinone in the human body, possessing numerous properties, with its most significant being its antioxidative and anti-inflammatory characteristics. The designation “10” signifies the number of isoprenyl units, accounting for its high solubility in fats, low polarity, and rapid penetration through the inner mitochondrial membrane [16]. It is noteworthy that CoQ10 exists in three forms—oxidized (ubiquinone), reduced (ubiquinol), and partially reduced (ubisemiquinone) [17]. These forms are presented in Figure 2. CoQ10 is endogenously synthesized from acetyl-CoA in the inner mitochondrial membrane, where it fulfils its primary biological role of electron transfer from complexes I and II to complex III [18]. This electron transfer contributes to the establishment of a proton gradient, ultimately leading to the efficient production of ATP. In addition, CoQ10 participates in the metabolism of other antioxidants, such as vitamin E and vitamin C [19]. It attains its highest concentration in tissues with high energy demands, such as muscles, kidneys, liver, and the heart, while its lowest concentration is found in the lungs and serum [16]. With age, the concentration of CoQ10 decreases in the heart, serum, and pancreas, accompanied by a shift in its redox status to oxidized from reduced. This process is associated with a decline in antioxidant capacity, thereby influencing the protection of lipoproteins and tissues in the blood [20]. Due to its antioxidative properties and significant role in ATP synthesis, CoQ10 plays a crucial role in meeting the energy demands of the cardiac muscle and other tissues. Among patients with CVD, studies have shown that CoQ10 concentrations are significantly lower compared to healthy individuals [21]. Over the past few years, numerous studies have been conducted to explore the impact of CoQ10 supplementation on the course of CVD.

### 2.2. Q10 Role’s in Heart Failure

ROS can induce cellular damage by interacting with protein centers, DNA, and cell membranes, as well as stimulate the proliferation of myocardial cells [22,23]. CoQ10 inhibits the initiation of lipid peroxidation processes, thereby exerting a protective influence on the cardiac muscle and other tissues [24]. In heart failure (HF), there is a decline in adenosine triphosphate (ATP) synthesis within the cardiac muscle, accompanied by an elevated generation of ROS and an alteration in calcium exchange, primarily attributed to ineffective electron transport chain function [16]. Referring to the meta-analysis of data investigating the impact of CoQ10 on HF, one can infer that supplementation of this compound demonstrates favorable effects on cardiac function, correlating with a reduction in mortality and hospitalization rates [25]. For instance, in 2014, a study was conducted, revealing that patients in the experimental group, receiving a daily dose of 300 mg of CoQ10, exhibited a reduced risk of major adverse cardiovascular events and experienced an improvement in disease symptoms compared to the control group [26]. Another study by Fladerer et al. from 2023 [27] found that while supplementation of the oxidized form of CoQ10 (ubiquinone) reduced the rate of cardiovascular death in patients with HF, the same effect was not observed for the reduced form (ubiquinol). Moreover, a recently published overview by Alarcón-Vieco et al. [25] analyzed preexisting data on CoQ10 supplementation in HF and found that it possessed a beneficial effect on heart function and mortality rates. CoQ10 was found to improve the ejection fraction (EF) by between 1.77% and 3.81%, which was an effect smaller than that exerted by traditional drugs, such as metoprolol (which increases EF by 7.4%) [25,28]. However, the data are inconsistent between different studies, the optimal supplementation dosage is unknown, and more trials evaluating the effect of CoQ10 in combination with drugs commonly used in HF are needed [25].

### 2.3. Q10 Role’s in Coronary Artery Disease and Dyslipidemia

Coronary artery disease (CAD) is a prevalent cardiovascular condition characterized by the formation of atherosclerotic plaques, leading to the narrowing of coronary artery lumens. This condition results in anginal symptoms, reduced tolerance to physical exertion, increased mortality, and a decline in the quality of life for affected individuals. Contributing factors to atherosclerotic plaque formation include oxidative stress and a state of chronic inflammation [29]. A systematic review conducted in 2019 revealed that CoQ10 supplementation contributes to a reduction in the levels of inflammatory markers among CAD patients. Additionally, higher concentrations of enzymes exhibiting antioxidative properties were detected, along with a decrease in malondialdehyde (MDA) levels, a marker of lipid peroxidation. These findings suggest that CoQ10 supplementation may be beneficial for CAD patients. However, to confirm these hypotheses, further research in this area is warranted [30]. One of the most significant risk factors for the development of CAD is dyslipidemia. It is widely acknowledged that elevated levels of LDL-C exert an atherogenic effect, whereas high concentrations of HDL-C prevent the formation of atherosclerotic plaques and serve as a favorable prognostic factor among patients with CVD. Studies have demonstrated that CoQ10 supplementation among CAD patients significantly reduces total cholesterol levels while concurrently increasing HDL-C concentrations [31]. On the other hand, in a randomized, double-blind, placebo-controlled study, it was demonstrated that CoQ10 supplementation did not significantly influence the lipid profile [32]. A recently published meta-analysis by Liu et al. [33] found that CoQ10 supplementation decreased total cholesterol, LDL-C, and triglycerides levels. Noteworthy, the meta-analysis also showed that the dosage of 400 to 500 mg/day exerted the greatest beneficial effect on total cholesterol [33]. Yet, investigations into the impact of CoQ10 on lipid concentrations have yielded inconclusive results, necessitating further research to conclusively elucidate the effects of supplementing this antioxidant on individual lipid fractions [34]. In 2022, a comprehensive study was conducted to investigate the effects of specific antioxidants and other micronutrients on CVD. In this study, it was demonstrated that CoQ10 reduces the concentration of specific lipoproteins by an average of 0.81 mmol/L for total cholesterol TC, 0.57 mmol/L for LDL-c, and 0.25 mmol/L for triglycerides TG. These results are significant compared to other studied micronutrients. Based on this study, it can be inferred that CoQ10 exhibits a more effective hypolipidemic effect than vitamin C, vitamin D, lycopene, resveratrol, quercetin, and isoflavones [35].

### 2.4. Q10 Role’s in Hypertension

Hypertension stands as a pivotal risk factor for the development of the majority of CVDs. Substantiated evidence suggests that CoQ10 diminishes arterial blood pressure and mitigates vasoconstriction, primarily through its anti-inflammatory and antioxidative properties [36]. In a systematic review conducted for primary prevention, the authors observed that CoQ10 supplementation led to a significant decrease in both systolic and diastolic blood pressure among patients who did not undergo lifestyle intervention [37]. In a randomized, double-blinded, controlled clinical trial conducted on a cohort of patients suffering from hyperlipidemia and myocardial infarction, supplementation with 200 mg of CoQ10 daily for 12 weeks resulted in a reduction in both systolic and diastolic blood pressure values [38]. Moreover, a recent meta-analysis by Zhao et al. [39] found that supplementation of CoQ10 was able to reduce systolic blood pressure by approximately −4.77 mmHg compared to the control group; however, it had no significant effect on diastolic blood pressure. The analysis also suggested that the CoQ10 dose of 100–200 mg/day had the best efficacy in decreasing systolic blood pressure [39]. However, the quality of the analyzed evidence was rated as moderate for systolic blood pressure and low for diastolic blood pressure according to the Grading of Recommendations, Assessment, Development, and Evaluation approach (GRADE) [39]. Moreover, CoQ10 reduced systolic blood pressure by an average of 3.55 mmHg and diastolic blood pressure by 0.92 mmHg. This indicates that CoQ10 was found to be more effective in lowering blood pressure than vitamin C, lycopene, isoflavones, and flavonoids, but less effective than resveratrol or L-arginine [35].

## 3. Polyphenols

### 3.1. Chemical Characteristics

**Figure 3:**
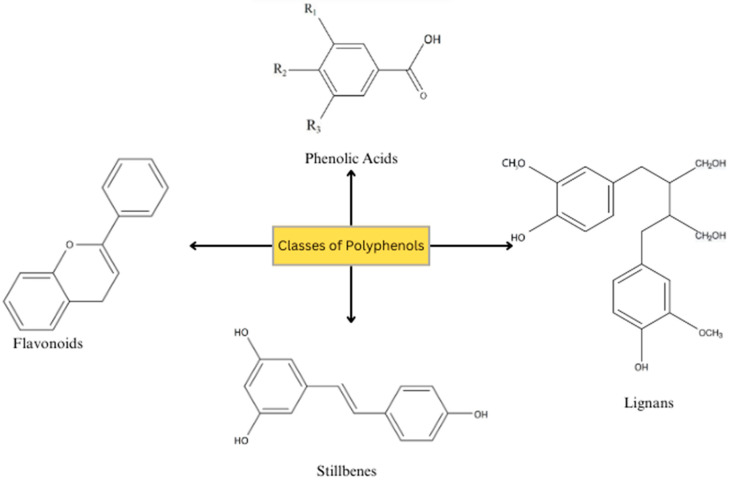
Chemical structures of the different classes of polyphenols. Extracted and modified from [36].

Polyphenols, chemically described as aromatic compounds featuring at least two phenolic groups, are bioactive compounds, predominantly found in foods such as fruits, grains, vegetables, chocolate, and beverages, such as coffee, tea, and wine, and they play a significant role in the prevention and treatment of CVD, offering protection against numerous chronic illnesses [40]. Polyphenols are typically classified based solely on the chemical structure of their aglycones, specifically focusing on the number of phenol rings and the binding chemical structures between them. All plant phenolic chemicals are derived from the same intermediate, phenylalanine, or a close predecessor, shikimic acid. They often occur in conjugated forms, with one or more sugar residues attached to hydroxyl groups; however, direct connections of the sugar (polysaccharide or monosaccharide) to an aromatic carbon are also possible. Associations with other molecules, such as carboxylic and organic acids, amines, and lipids, as well as coupling with other phenols, are prevalent. Polyphenols can be categorized into distinct categories based on the amount of phenol rings they contain and the structural components that tie these rings together. Consequently, they are categorized into four groups: phenolic acids, stilbenes, lignans, and flavonoids (Figure 3) [41]. In our review, we aimed to offer an analysis of the most recent scientific insights into the effects of polyphenols on various cardiovascular diseases, with a specific focus on flavonoids and stilbenes.

The advantageous effects of polyphenols on cardiovascular health are attributed to their direct antioxidant action, which involves scavenging ROS and reactive nitrogen species [42]. Nonetheless, increasing evidence indicates alternative mechanisms that could also explain the observed decrease in CVD risk linked to the intake of polyphenols. These mechanisms include direct anti-inflammatory effects, modulation of intracellular signaling pathways and gene expression, maintenance of nitric oxide homeostasis, and antiplatelet aggregation capacity. However, polyphenols differ in their site of absorption in humans. Some of the polyphenols are well absorbed in the gastrointestinal tract, while others in the intestine or other parts of the digestive tract. Flavonoids, except flavanols, exist in glycosylated forms in foods. Absorption in the stomach is unclear, but some flavonoids, such as quercetin, can be absorbed at the gastric level. Glucosides may be transported into enterocytes by SGLT1 and hydrolyzed by β-glucosidase. Proanthocyanidins, due to their polymeric nature and high molecular weight, may limit absorption through the gut barrier [43].

### 3.2. Flavonoids

Flavonoids are secondary metabolites that mostly consist of a benzopyrone ring with phenolic or polyphenolic groups at various locations [44]. They are mostly present in fruits, herbs, stems, grains, nuts, vegetables, flowers, and seeds. The presence of bioactive phytochemical elements in these various plant sections provides them with therapeutic potential and biological activity. More than 10,000 flavonoid compounds have been isolated and identified. Most flavonoids are extensively recognized as medicinal agents. These are naturally generated via the phenylpropanoid pathway, with bioactivity determined by the absorption mechanism and bioavailability. Flavonoids have been employed in natural colors, cosmetics and skin care products, and anti-wrinkle creams [45]. However, these polyphenols are most commonly used in medicine.

**Figure 4:**
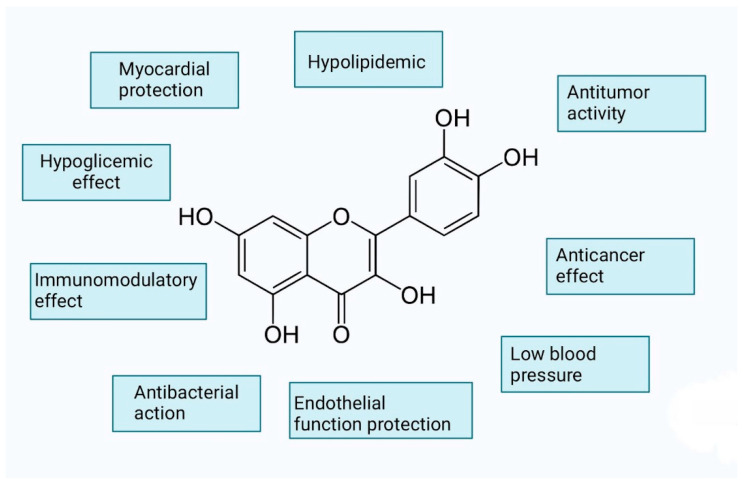
Flavonoids’ properties.

Flavonoids have been widely employed as anticancer, antibacterial, antiviral, antiangiogenic, antimalarial, antioxidant, neuroprotective, antitumor, and anti-proliferative drugs (Figure 4) [46]. Apple peel preparations high in flavonoids inhibit acetylcholinesterase (ACE) in vitro and are an efficient antihypertensive drug [47]. It also protects against cardiometabolic illnesses and improves cognitive function with age. These compounds act as potent antioxidants as they reduce low-density lipoprotein (LDL) cholesterol oxidation, modulate cell signaling pathways, and reduce platelet aggregation [44]. Epidemiological studies show that flavonoids have health benefits, which are often linked to their antioxidant activity. In vitro investigations, however, revealed that many flavonoids hinder organification in thyroid cells and follicles. Studies in vivo and in vitro using synthetic and natural flavonoids revealed T4 displacement from transthyretin, resulting in changes in thyroid hormone availability in tissues. Radioactively tagged flavonoids appeared to be quickly removed from the body, primarily by fecal excretion. In pregnant rats, synthetic flavonoids pass the placenta and accumulate in the fetal compartment, including the brain. As a result, consuming excessive amounts of flavonoids is not recommended [48].

They are categorized into several categories based on their chemical structure, level of unsaturation, and carbon ring oxidation. Flavonoids are classified into many subgroups: anthoxanthins (flavanone and flavanol), flavanones, flavanonols, flavans, chalcones, anthocyanidins, and isoflavonoids. Each of these flavonoids is commonly found in nature. An increased consumption of flavonoid-rich foods provides a range of health advantages, and because these natural substances have beneficial benefits on human health, there has been an increased attempt to separate them from diverse plants. Citrus fruits are an excellent source of flavonoids. Oranges, lemons, and grapes contain two flavonoids called narigenin and hesperetin [49]. Mulberry contains anthocyanins and quercetin glycosides, both flavonoids.

#### 3.2.1. Anthoxanthins (Flavanone and Flavanol)

Kaempferol is a flavonoid with anti-inflammatory properties [45], which prevent atherogenesis in THP-1 macrophages via regulating monocyte-to-macrophage differentiation, pro-inflammatory gene expression, monocyte mobility, IFN-γ-mediated inflammation, and cholesterol export. Kaempferol might be used to lower the risk of atherosclerotic diseases by altering the expression of disease-related genes, perhaps leading to the creation of a new additional treatment for CVD [45]. Kaempferol, a phytochemical rich in plant-derived foods, is commonly consumed as a phytochemical in a well-balanced diet due to its anti-inflammatory and anti-atherosclerotic effects. Its mechanism of action involves inhibiting the production of pro-inflammatory genes, MCP-1 and ICAM-1, involved in atherosclerosis progression. Kaempferol, along with other flavonoids, possesses diverse biological effects that can be either beneficial or harmful depending on the situation. Research suggests that kaempferol may have mutagenic and genotoxic properties, primarily because it can induce oxidative stress in laboratory conditions. This occurs when kaempferol interacts with free radicals, acting either as an antioxidant by neutralizing them or as a pro-oxidant, leading to the production of reactive oxygen species. Moreover, kaempferol’s pro-oxidant activity is associated with its ability to reduce metal ions, which can further enhance oxidative damage by generating hydroxyl radicals [50].

Oxidative stress, which results from an imbalance between cellular oxidants and antioxidants, can lead to damage to proteins, lipids, and DNA, triggering inflammatory reactions. Kaempferol demonstrates strong antioxidant properties by scavenging various reactive oxygen and nitrogen species, inhibiting lipid peroxidation, and safeguarding against oxidative damage in various cell types. Furthermore, it regulates cellular responses to inflammation by affecting the activity of redox-sensitive transcription factors, such as Nrf-2, thus enhancing cellular defense mechanisms against oxidative stress-induced harm [51].

Kaempferol inhibits ERK1/2 by downregulating cytokine receptors, reducing cardiac failure and hypertrophy. Additionally, CYP1B1 plays a crucial role in the metabolism of various xenobiotics, arachidonic acid, estrogen, and cholesterol, which are important in the etiology of CVD, including heart hypertrophy and hypertension [52].

Rutin is a flavonoid molecule that has anti-inflammatory and vascular protective properties. The available data show that rutin improves cardiovascular structure and function in rats, including hypertrophy, inflammation, and fibrosis. It prevents atherosclerosis by lowering total cholesterol, triglycerides, non-esterified fatty acids, and insulin levels in rat blood. Rutin improves metabolic disorders and slows the premature aging of vascular smooth-muscle cells, lowering the burden of atherosclerosis and stabilizing plaques, and may have a role in the treatment of diabetic atherosclerosis [45]. The study looks at the function of rutin in vascular calcification, which is a possible risk factor for CVD [53]. Air pollution affects around 80% of the Chinese population, and prolonged exposure to air pollution is highly related with atherosclerosis and CVD. Winter coal production in Taiyuan, a hilly region in northern China, contributes to the high organic content, concentration, and major heavy metal components in winter PM2.5. Vascular calcification is an important pathophysiological substrate for CVD development.

The findings revealed that PM2.5 exposure accelerated vascular calcification, which was linked to the activation of oxidative stress and OPG/RANKL in vivo and in vitro. Rutin supplementation reduced PM2.5-induced vascular calcification via the OPG/RANKL signal pathway and ROS production. Following PM2.5 exposure, RANKL expression rose while OPG expression decreased, showing that PM2.5 promoted vascular calcification through the OPG/RANKL signaling pathway both in vivo and in vitro. PM2.5 exposure activated the nuclear translocation of NF-κB, which plays a crucial role in pro-inflammatory responses. This activation is critical for RANKL-mediated osteogenic differentiation of smooth-muscle cells, which contributes to vascular calcification [54]. PM2.5 exposure also causes OS, which results in oxidative damage. PM2.5 exposure boosts the expression of NOX4 and p22phox, two components of the superoxide-producing NAPDH (reduced form of nicotinamide-adenine dinucleotide phosphate) oxidase system, which is also linked to vascular calcification [45].

#### 3.2.2. Flavanones

Viscosine is a flavonoid extracted from *Dodonea viscosa* that exhibits anti-inflammatory, antipyretic, and antioxidant effects. Baicalin is a flavonoid found in medicinal plants, such as *Scutellaria baicalensis* Georgi and *Oroxylum indicum*. This flavonoid has antioxidant and anti-inflammatory properties and is used to treat conditions such as asthma, liver and kidney disease, inflammatory bowel disease, carcinogenesis, and CVD. Chrysin, a flavonoid, also exhibits anti-inflammatory and antioxidant properties [55].

#### 3.2.3. Chalcones

Chalcones are the natural precursors to flavonoids and iso-flavonoids. They are present in many plants and vegetables and have a wide range of biological functions. Chalcone is an aromatic ketone and enone that can trigger the nuclear factor erythroid 2-related factor (2NRF2) pathway [56]. Several novel dihydroxy chalcones were synthesized and investigated for their ability to lower ROS and oxidative stress, acting as an anti-ischemic stroke agent by activating the KEAP1/NRF2/ARE pathway [56]. Rat models were utilized to study cerebral ischemia-reperfusion injury (CIRI) after a stroke. Not only did these compounds efficiently protect neuron-like PC12 cells from H_2_O_2_-induced oxidative damage, but they also showed neuroprotective capabilities against ischemia/reperfusion-related brain injury in animals. These inflammatory genes are activated after a stroke. Stem cells are capable of producing anti-inflammatory substances. Following an ischemic stroke, endothelial progenitor cells (EPCs) exert a direct impact on the inflammation-associated stroke vasculome [57]. Fisetin, a flavonoid, inhibits LPS-induced TNFα production and decreases nuclear factor jB activation, making it a neuroprotective and anti-inflammatory medication for post-ischemia damage in vitro. Focal cortical dysplasia (FCD) develops when neurons in the brain do not migrate correctly during their development in utero. Rutin, a naturally occurring flavonoid, has been used in animal studies to treat FCD. Motor neurons recovered considerably when administered 50 mg/kg. This might be employed as a medicinal drug in the future [58].

### 3.3. Stilbenes

Resveratrol, also known chemically as 3,4′,5-trihydroxystilbene, is a naturally occurring stilbene, a bioactive molecule with pleiotropic biofunctions, naturally produced by many plants, primarily grapes and peanuts. This polyphenol has been presented in numerous studies as a substance with a wide range of beneficial effects on human health, including its impact on CVD [59,60]. The pleiotropic effect of resveratrol arises from its ability to interact with diverse targets, including kinases, receptors, and signaling molecules [61].

#### 3.3.1. Resveratrol’s Impact on Oxidative Stress, Inflammation, and NO Synthesis

Early research demonstrates that resveratrol effectively reduces oxidative stress by inhibiting human LDL oxidation and lipid peroxidation. In mice, resveratrol supplementation decreased lethality from lipopolysaccharide (LPS) exposure and enhanced antioxidative enzyme activities in the myocardium and aorta. Resveratrol exhibits notable anti-inflammatory effects by inhibiting COX-1, thus reducing pro-inflammatory eicosanoid synthesis. Furthermore, resveratrol enhances vasculoprotective nitric oxide (NO) synthesis through eNOS activation, which is mediated by estrogen receptors, MAPK signaling, and SIRT1 activation. Animal studies confirmed these findings, with resveratrol improving endothelial functionality in hypercholesterolemic rabbits and enhancing eNOS and NO levels in the plasma [59]. Research findings indicate that resveratrol reduces ROS production and lowers levels of oxidative stress and blood pressure by enhancing the capability of redox proteins to regulate redox balance within cells [62]. Moreover, by activating SIRT1, it can augment the activity of endothelial nitric oxide synthase (eNOS), thereby suppressing the expression of the angiotensin II receptor. This impedes vessel constriction induced by angiotensin II, leading to vasodilation and a reduction in blood pressure. Studies unequivocally suggest that resveratrol supplementation could, therefore, influence the prevention and treatment of hypertension [63,64].

#### 3.3.2. Resveratrol and Lipid Oxidation

Joao Tomé-Carneiro et al. evaluated the impact of a grape supplement containing 8 mg of resveratrol on oxidized LDL, apolipoprotein B (ApoB), and serum lipids in patients undergoing statin therapy as part of primary prevention of CVD. Patients with CVD were administered GE-RES, GE, or a placebo in combination with statins, commonly prescribed drugs for CVD [65]. The GE-RES formulation was created by blending 8 mg of resveratrol with grape extract (GE). Within the GE-RES group, a notable reduction in LDLc, ApoB, and the ratio of oxidized LDL/ApoB was observed. Conversely, in the GE group, only a decrease in LDLc was evident [65]. Additionally, GE-RES significantly reduced hsCRP levels, which correlated well with the decrease in TNF-α and plasminogen activator inhibitor type 1 (PAI-1) levels [66]. Considering PAI-1′s involvement in the pathogenesis of obesity, diabetes, and CVD, it could be deemed an attractive target for addressing these conditions [67].

In a comprehensive cross-sectional study, called PREDIMED, which focused on individuals at a heightened risk of CVD, findings revealed that a high level of resveratrol metabolite in urine correlated with a decreased risk of CVD. Moreover, the combination of moderate wine consumption with resveratrol supplementation was linked to enhancements in the blood lipid profile, fasting blood glucose levels, and heart rate. Nevertheless, it is worth noting that resveratrol on its own may only lead to a reduction in fasting blood glucose levels [68].

#### 3.3.3. Resveratrol on Atherosclerosis

Resveratrol, as many different polyphenols, revealed many cardiovascular benefits. (Table 1). Resveratrol has demonstrated significant anti-atherosclerotic effects by inhibiting the dysregulated and excessive proliferation of vascular smooth-muscle cells (VSMCs), a key contributor to atherosclerosis and restenosis post-vascular surgery. Studies indicate that resveratrol suppresses VSMC proliferation induced by advanced glycation end-products (AGEs), oxidized low-density lipoprotein (ox-LDL), and endothelin-1 (ET-1). Mechanistically, these effects are associated with the suppression of MAPK ERK1/2 and a reduction in ROS and H_2_O_2_ production. Additionally, resveratrol attenuates VSMC proliferation induced by hypoxia and homocysteine, while also reducing collagen synthesis and promoting the expression of protective factors. Resveratrol’s vasculoprotective benefits extend to inhibiting platelet aggregation, further mitigating the atherosclerosis risk. In animal models, resveratrol supplementation has been shown to reduce platelet aggregation and enhance endothelial function, highlighting its potential as a therapeutic agent in managing atherosclerosis [59].

#### 3.3.4. Resveratrol and Protective Effect for Heart Injury

Resveratrol’s positive effects on cardiomyocytes in an ischemia-reperfusion paradigm include superoxide suppression, potassium channel activation, and increased endothelium-dependent vasodilation. In rats fed a high-cholesterol diet and exposed to an experimentally induced myocardial infarction, oral resveratrol supplementation altered specific cardiologic parameters, such as ejection fraction and fractional shortening, and promoted neovascularization in the damaged myocardium. Furthermore, feeding rats resveratrol for three weeks protected them against ischemia/reperfusion damage and restored an aberrant microRNA pattern. Resveratrol improved survival, hemodynamics, and energetics in rats in a model of hypertension that leads to heart failure [53].

Numerous studies also demonstrated significant cardioprotective properties of resveratrol and its potential therapeutic effects in patients with CHD and post-myocardial infarction [60,61]. Chekalina et al. demonstrated an improvement in both systolic and diastolic function of the left ventricle as well as left ventricular ejection fraction in patients with CHD following treatment with resveratrol [52]. Following myocardial infarction, patients exhibited improvements in endothelial function and left ventricular diastolic function, as well as reduced LDL levels, even with very low doses of resveratrol (10 mg/day; 3 months) [63].

In another study on patients diagnosed with stable angina pectoris, a combination therapy of resveratrol (20 mg/day) and calcium fructoborate (112 mg/day) was administered. The study evaluated the levels of hsCRP and brain natriuretic peptide (BNP), recognizing prognostic markers for inflammation and left ventricular function in cardiovascular patients. The combined treatment significantly decreased levels of hsCRP and BNP, credited to the synergistic effect of both agents [62].

It is worth mentioning a relatively new double-blind clinical trial that assessed the potential benefits of RES in patients with symptomatic systolic HF. This study enrolled sixty patients with New York Heart Association (NYHA) class II–III HF who were randomly assigned to one of two groups: (1) receiving RES supplementation at a dose of 100 mg/day for 3 months or (2) receiving a placebo. All patients were receiving maximally tolerated medical therapy for HF at the time of study inclusion, in accordance with the guidelines. At the end of the study, improvements in systolic and diastolic cardiac function and global cardiovascular burden were observed in the RES group, along with reductions in serum levels of biomarkers (NT-proBNP and galectin-3) and inflammatory cytokines (IL-1 and IL-6); furthermore, the treatment group exhibited a higher exercise capacity and reported better quality of life [69].

#### 3.3.5. Resveratrol and Limitations

Although resveratrol shows therapeutic potential, its low bioavailability after oral administration is a significant limitation [70,71,72]. Studying the metabolism of resveratrol reveals that it is rapidly absorbed after ingestion. Despite this quick absorption, its levels remain very low due to rapid metabolism [70]. Gambini et al. conducted a study in which, after the oral consumption of 25 mg of resveratrol, they observed a peak serum concentration of less than 10 ng/mL after 0.5 h [71]. Resveratrol is also rapidly eliminated from the body. Approximately 77–80% of ingested resveratrol is absorbed in the intestines, with 49–60% of that being excreted in the urine. Consequently, 75% of the ingested resveratrol is removed from the body. The remaining portion is metabolized, with the maximum concentration of free resveratrol being only 1.7–1.9% [70].

Various strategies have been described to improve the pharmacokinetic properties and beneficial effects of resveratrol. These methodological approaches include nanoencapsulation in lipid nanocarriers or liposomes, nano-emulsions, micelles, incorporation into polymeric particles, solid dispersions, and nanocrystals [73]. One of the methods used in an in vitro study aiming to improve resveratrol’s bioavailability was drug yeast encapsulation. Despite the absence of in vivo studies, the authors concluded that the rapid metabolism and elimination contributing to the poor bioavailability of resveratrol could be partially mitigated through yeast cell encapsulation technology [74].

The focus of recent research is on innovative resveratrol delivery systems based on lipid nanoparticles. These sophisticated controlled-release systems are specifically engineered to transport and shield this bioactive compound from degradation. By doing so, they significantly enhance its physical stability and boost its oral bioavailability. Lipid nanoparticles, which are submicron colloidal carriers, are made from biodegradable and biocompatible lipids. These materials are widely acknowledged for their safety and appropriateness in encapsulating lipophilic and poorly water-soluble substances, such as resveratrol. This encapsulation process facilitates the efficient absorption of resveratrol when taken orally [75]. Neves et al. developed resveratrol nano-delivery systems based on solid-lipid nanoparticles and nanostructured lipid carriers. The research revealed that resveratrol was released only minimally over several hours from both nanocarrier systems, indicating that these lipid nanoparticles are highly stable. Simulations of the gastrointestinal tract in vitro showed that resveratrol largely remained associated with the lipid nanoparticles even after exposure to digestive fluids. Consequently, these delivery nano-systems are deemed effective for oral administration, as they protect the encapsulated resveratrol and facilitate its controlled release upon absorption, thereby improving the bioavailability of the compound [75].

Resveratrol exhibits a different effective dosage range in vitro (micromolar range in cell culture media) compared to its in vivo bioavailability (nanomolar range in the blood), complicating the determination of the biologically effective concentration for human supplementation. Concerns have been raised about achieving the effective in vitro concentrations in vivo. Therefore, the actual biologically effective concentration range of resveratrol in vivo remains undetermined. Although human tissue and organ levels are still being studied, evidence from rodent studies shows that resveratrol can accumulate in specific tissues or organs at concentrations comparable to those used in many in vitro experiments [60].

In conclusion, current literature shows that resveratrol possesses substantial therapeutic potential in addressing CVD. Despite recent advances in our understanding of how resveratrol affects CVD, and in particular CHD, further research is still required to appreciate what clinical significance this has at a population-based level.

**Table 1:** The source and role of different polyphenols and their cardiovascular benefits.

Polyphenol	Source	Cardiovascular Benefits
Resveratrol	Grapes, red wine, berries	Improves endothelial function, reduces blood pressure, anti-inflammatory effects, decreases LDL oxidation, and inhibits platelet aggregation [76].
Epicatechin	Dark chocolate, green tea	Enhances endothelial function, improves blood flow, lowers blood pressure, reduces oxidative stress, and improves cholesterol profiles [77].
Quercetin	Apples, onions, berries	Anti-inflammatory effects, reduces blood pressure, improves endothelial function, and decreases LDL oxidation [78].
Catechins	Green tea, cocoa, apples	Antioxidant effects, improves endothelial function, reduces blood pressure, lowers cholesterol levels, and enhances nitric oxide availability [79].
Anthocyanins	Berries, red grapes, red cabbage	Reduces oxidative stress, anti-inflammatory effects, improves endothelial function, lowers blood pressure, and enhances nitric oxide production [48].
Flavonols	Tea, onions, kale	Reduces blood pressure and has anti-inflammatory effects [80].

## 4. Carotenoids

### 4.1. Chemical Characteristics

Carotenoids are organic pigments, belonging to the tetraterpenes family, that can be found in many foods, such as fruits, vegetables, and fish [81,82]. The typical human diet contains around 40 out of more than 600 carotenoids, and only 20 carotenoids have been found to be present in human blood and tissues [81,83].

Most carotenoids are composed of a central carbon chain of alternating single and double bonds and different cyclic or acyclic end groups [84]. The group can be classified into provitamin A (namely β-carotene, α-carotene, and β-cryptoxanthin) and non-provitamin A compounds [84]. Based on which functional group they contain, carotenoids can also be divided into xanthophylls (such as lutein or zeaxanthin), which contain oxygen and carotenes (such as α-carotene, β-carotene, and lycopene) with only a hydrocarbon chain and no functional group [85].

One of the significant features of the carotenoids is their strong coloration, which is a consequence of light absorption in the presence of a conjugated chain [86,87]. For example, β-carotene, α-carotene, and β-cryptoxanthin are orange, lutein is yellow, and lycopene is red [86].

### 4.2. Antioxidant Capabilities

Carotenoids possess beneficial antioxidant properties because of conjugated double bonds, and they are able to accept electrons from reactive species and neutralize free radicals [88]. Carotenoids act as ROS scavengers—they are capable of trapping peroxyl radicals and quenching the singlet oxygen [89]. The scavenging occurs in three steps: electron transfer (oxidation and reduction), hydrogen abstraction, and addition [90]. Moreover, carotenoids are involved in the process of deactivation of electronically excited sensitizer molecules, which partake in the production of singlet oxygen and radicals [91]. The carotenoids’ efficacy in quenching the singlet oxygen is dependent on the number of conjugated double bonds present in the molecule; therefore, the most effective quencher is the open-ring carotenoid lycopene [84].

### 4.3. Carotenoids in the Human Body

Carotenoids are absorbed from food into gastrointestinal mucosal cells, formed into the chylomicrons, and released into the lymphatic system, and then they bind the lipoprotein at the liver and are released into the bloodstream [92,93,94]. Carotenoids are primarily accumulated in adipose tissue and the liver, but also in the cervix, lungs, skin, and eyes [95,96,97]. Particularly, carotenoids are stored in greater amounts in tissues that contain more low-density lipoprotein receptors (LDL-R), probably as a result of a non-specific uptake by lipoprotein carriers [98]. The positive health effect of carotenoids is primarily dependent on their antioxidant activities [98,99]. For instance, they act as photo-protectants and are involved in protecting the skin and eyes [98]. Their antioxidant abilities have also been suggested to be one of the mechanisms of their cardiovascular beneficial qualities [98].

### 4.4. Carotenoids and Cardiovascular Diseases

Oxidatively modified LDL plays a major role in the initiation and promotion of atherosclerosis and coronary heart disease (CHD) [100]. Free radicals, which cause LDL oxidation, partake in the production of foam cells and, thus, promote atherogenesis [101]. Therefore, it has been speculated that antioxidants, which hamper LDL oxidation, may exhibit a protective effect against CHD [82]. It has been observed that patients with CAD presented lower plasma levels of oxygenated carotenoids compared to the general population [102]. Moreover, it has been found that the reduced levels of lutein, zeaxanthin, and β-cryptoxanthin correlated with smoking, high body mass index, and low high-density lipoprotein cholesterol (HDL-C) [102]. Studies have shown that low plasma levels of lycopene were associated with subclinical atherosclerosis (understood as an increase in intima-media thickness of the common carotid artery) [103].

Furthermore, evidence has shown that higher serum levels of carotenoids were correlated with a decreased risk of elevated serum NT-proBNP levels, which might indicate that carotenoids can partake in preventing cardiac overload [104]. It has also been suggested that high plasma levels of β-cryptoxanthin and lutein might be linked to a lower risk of acute myocardial infarction [105]. Moreover, a study by Akbaraly et al. [106] confirmed that high plasma carotenoid levels correlate to a reduced risk of dysglycemia. The CARDIA study [107] found that the risk of developing hypertension was lower in individuals with higher concentrations of carotenoids.

A study by Xu et al. [108] showed that serum carotenoids were associated with a reduced risk of atherosclerosis and inversely associated with inflammatory cytokines. Therefore, it is speculated that carotenoids can reduce the risk of CVD by improving the lipid profile, preventing lipid peroxidation, contrasting vascular wall inflammation, stabilizing membrane properties, and thus acting in opposition to the pathophysiologic steps of atherosclerosis [109].

Since β-carotene and lycopene are carotenoids primarily transported in LDL, it has been suggested that they may be the most effective in protecting LDL from oxidation; however, many other carotenoids have been studied as well [110].

### 4.5. β-Carotene

β-Carotene is a quencher of singlet oxygen and a radical-trapping antioxidant [111]. It is able to inhibit LDL oxidation and, as a result, reduce its degradation by macrophages; thus, it is speculated that it may prevent atherosclerosis [111]. However, a study by Shaish et al. [112] showed that, while all-trans β-carotene inhibited atherogenesis in hypercholesterolemic rabbits, the effect may be separated from the LDL resistance to oxidation and may depend on stereospecific interactions with retinoic acid receptors in the artery wall. A study by Di Tomo et al. [113] found that β-carotene and lycopene were able to significantly decrease tumor necrosis factor-α-induced inflammation in human endothelial cells, meaning that they exerted a positive effect against CVD by opposing inflammatory oxidative stress.

An observational epidemiologic study by D’Odorico et al. [114] provided evidence that β-carotene had a protective effect on early atherogenesis and CVD, whereby the antioxidant properties countered the development of atherosclerotic lesions. Studies on the population of middle-aged Finnish men found that low plasma levels of β-carotene increased the risk of CVD mortality as well as sudden cardiac death [115,116]. Moreover, a study by Street et al. [117] found that decreased serum levels of β-carotene were associated with an increased risk of myocardial infarction in the population of smokers.

However, some clinical trials have not found β-carotene supplementation to have any beneficial effect on CVD or death incidence [118,119]. It is speculated that the conflicting results may be a result of differences in the trials’ performances or the form of dietary β-carotene; nevertheless, the beneficial effect of β-carotene remains uncertain [109].

### 4.6. Lycopene

Lycopene has been found to exert an anti-atherogenic effect, which is linked to its anti-inflammatory capabilities, improved lipid homeostasis, and inhibition of IL-1 secretion [97,120]. Lycopene is also able to positively influence nitric oxide levels, which contribute to vasodilatation, therefore, slowing the progression of atherosclerosis [121].

A study by Alvi et al. [122] showed that lycopene was able to target the expression of the liver genes PCSK-9 and HMGR, which resulted in an increase in LDL receptor activity and, therefore, a lowering of hypercholesterolemia. Lycopene was also found to improve the LDL/HDL ratio, reduce the accumulation of cholesterol in the aorta, lower the synthesis of dysfunctional HDL, and inhibit vascular smooth-muscle cell proliferation and foam cell formation, and thus, to have a beneficial effect on the initial stages of atherosclerosis [123,124,125].

Several studies have shown that lower blood levels of lycopene were correlated with an increased risk of atherosclerotic lesions and an increased risk of acute coronary events or stroke [126,127,128]. Consequently, high lycopene levels were found to be associated with a lower risk of CVD in women [129]. Moreover, a study by Kim et al. [130] found that an increased dietary intake of lycopene correlated with reduced hypertension in overweight and obese individuals, while another study by Han et al. [131] suggested that a higher serum concentration of lycopene was associated with a reduced risk of mortality in the population with metabolic syndrome. Another study by Polidori et al. [132] found that in the population with HF, the left ventricular ejection fraction was significantly and positively correlated with plasma lycopene levels, and increased consumption of antioxidant micronutrients, such as lycopene, might help in achieving cardiovascular health.

However, some studies have shown that lycopene exerted no beneficial effect on CVD risk [133,134]. Yet, an epidemiologic follow-up study by Ito et al. [135] found that high serum levels of lycopene were indeed associated with low hazard ratios for cardiovascular mortality. A systematic review and meta-analysis of 43 studies by Tierney et al. [136] found that the data on lycopene’s efficacy in improving cardiovascular risk were conflicting.

Role of different carotenoids in preventing CVD is presented in Table 2. 

## 5. Novel Experimental Antioxidant Therapies

Imbalanced ROS production during intense oxidative stress leads to exacerbation of pathophysiological processes in humans [137]. In many CVDs, such as myocardial infarction, hypertension, atherosclerosis, myocardial hypertrophy, HF, and restenosis after angioplasty or venous bypass, excessive ROS production plays an important role in their development [138]. There is, therefore, great hope that antioxidants and substances with antioxidant activity might minimize the negative effects of ROS and, at the same time, help to improve the prognosis of patients with CVD.

Novel antioxidants include compounds that are activators of endogenous antioxidant defense systems, compounds that are inhibitors of oxidative stress generation, and compounds that enable functional repair of ROS-induced damage.

Activators of endogenous antioxidant defense systems include the activator NRF2, which is a basic transcription factor that recognizes an enhancer of the antioxidant response element. Its reduced expression and activity have been shown to predispose to the development of hypertension or atherosclerosis [139]. The drug targeting NRF2 is dimethyl fumarate (DMF) [140]. Experiments have shown that it reduces infarct size after ischemia/reperfusion injury [140] and has a protective role against cardiomyocytes after this injury [141]. DMF also reduced the development of atherosclerosis in an apolipoprotein-E-deficient study [142]. DMF was shown to prevent endothelial dysfunction [143].

Inhibitors of oxidative stress generation include drugs targeting Xo, NOX, and MPO, among others. Allopurinol is an Xo inhibitor that has shown beneficial effects in clinical trials in hypertension, HF, and ischemia/reperfusion injury by reducing oxidative stress in endothelial cells [144]. In a meta-analysis evaluating its effects on blood pressure, it showed a moderate reduction in systolic and diastolic blood pressure in patients [145], which may be related to its ability to improve endothelial function [146]. It was also shown to reduce in-hospital mortality and cardiac complications in patients undergoing primary percutaneous coronary intervention or coronary artery bypass grafting [147,148]. Allopurinol has been shown to improve myocardial oxygen consumption and myocardial blood flow [149]. It may improve exercise capacity in patients with chronic HF [146]. The use of allopurinol in patients with ischemic cardiomyopathy has been associated with a significant improvement in left ventricular ejection fraction and a reduction in left ventricular end-systolic volume [150].

GKT137831 is a NOX inhibitor in clinical trials. When used in ApoE knock-out mice, the drug had a potent anti-atherosclerotic effect [151]. It also improved cardiac function after ischemia/reperfusion injury [152]. In contrast, MPO inhibitors led to changes in the composition of atherosclerotic lesions and cardiac remodeling in mouse studies [153].

Compounds that enable functional repair of ROS-induced damage include compounds that affect nitric oxide–cyclic guanosine monophosphate (NO-cGMP) signaling, including HNO donors, such as CXL-1427, L-citrulline, or L-arginine [154]. In clinical trials, CXL-1427 showed a favorable safety profile and hemodynamic effects in HFrEF patients [155]. For L-arginine and L-citrulline, meta-analyses of randomized clinical trials showed that oral administration of these compounds was associated with reductions in systolic and diastolic blood pressure [156].

### 5.1. miRNA

miRNAs are involved in the oxidative stress response and play a key role in its regulation, and therefore represent important compounds for therapeutic intervention against various pathological conditions. Future clinical applications of miRNAs in the treatment of CVD include the use of induction (restoring miRNAs that have lost function) as well as inhibition of miRNA expression [157]. In addition, modifications or carriers must be used to increase the stability and bioavailability of the molecules [158,159].

During hypoxia, miRNA-210 levels are significantly increased, leading to improved cardiac function by promoting angiogenesis and inhibiting cardiomyocyte apoptosis [160,161,162]. Direct injection of miRNA-210 into the myocardium in an animal model of myocardial infarction resulted in improved myocardial function [163]. Circulating miRNA-210 levels were found to be significantly associated with mortality in patients with acute coronary syndrome [164].

The most abundant of these, which also plays a key role in muscle cell differentiation and proliferation, is miRNA-1. It is a regulator of cardiomyocyte growth and a pro-apoptotic factor in anemic myocardium, as observed in diseases such as hypertrophy, myocardial infarction, and arrhythmias [165,166,167]. Studies have shown that its overexpression is associated with increased ROS and decreased SOD production [168]. Furthermore, H_2_O_2_ increased miRNA-1 in cardiomyocytes in a rat model [168]. Increased miRNA-1 was associated with a significant reduction in infarct size [169] and its serum levels correlated with circulating troponin T, suggesting that it may be used as a biomarker for myocardial infarction [170,171]. Administration of miRNA-1 to mice after myocardial infarction improved myocardial function [172].

It has been observed that some miRNAs may play an important role in regulating atherosclerotic plaque formation. miRNA-133 was elevated in the presence of symptomatic atherosclerotic plaques and in patients with CVD [173,174]. Its inhibition mainly targets NOS and may prevent vascular endothelial dysfunction [175]. Regulation of endothelial NOS expression may also be influenced by miRNA-92a [176], which further reduces plaque inflammation and increases plaque stability by promoting endothelial cell proliferation and angiogenesis [177]. In the case of miRNA-206, which modulates VEGF expression, there is inhibition of viability and invasion and increased apoptosis of endothelial progenitor cells in patients with CAD [178]. In contrast, inhibition of miRNA-377 had a protective effect. It reduced myocardial fibrosis and improved myocardial function [179]. The functions of the miRNAs mentioned above are shown in Table 3.

### 5.2. Nanoparticles

Nanoparticles could have a groundbreaking impact on the treatment of CVD due to their size and properties, which allow them to be easily modified [180]. Among other things, research is testing H_2_O_2_-responsive nanoparticles that would target the site of ischemia/reperfusion injury in the myocardium. Such particles have shown potent anti-inflammatory and anti-apoptotic effects in various animal models, leading to a reduction in further organ damage [181]. Nanoparticles with antioxidant properties can be used in anticoagulant therapy, alongside existing thrombolytic agents, due to fibrin aggregation and increased H_2_O_2_ levels in thrombi [182]. This involves imaging the thrombus and then inhibiting its formation by scavenging H_2_O_2_. Other studies have also used nanoparticles to reduce oxidative stress by modifying its production or removal system. In one study, nanoparticles linked to the small-interfering RNA (siRNA) NOX2 were injected directly into the heart muscle of mice after a heart attack, resulting in improved heart function [183]. Studies are also focusing on the protective role of SOD, using nanoparticles capable of carrying it. When injected into the ischemic area of the myocardium in a rat model of ischemia/reperfusion, it resulted in reduced myocyte apoptosis and improved myocardial function [184]. Nanoparticles designed to carry *N*-acetylcysteine showed effective attenuation of myocardial fibrosis in rat models of ischemia/reperfusion [185]. In addition, selenium-based nanoparticles showed improved biological effects in ischemic cardiomyocytes due to their ROS-quenching properties [186]. In studies, nanoparticles attenuated ROS-induced inflammation and cell apoptosis in macrophages by scavenging intracellularly generated ROS, effectively preventing foam cell formation and reducing internalization of oxidized LDL [187]. Potential roles of nanoparticles in the prevention and treatment of CVDs are presented in Table 4.

The use of antioxidant therapies is mainly based on enhancing their effects through supplementation. This is related to the continuing failure of therapies designed to modulate oxidative stress. This may be due to non-selective modulation of ROS, which would interfere with physiological ROS-dependent signaling pathways, or to insufficient efficacy of modulation [134]. Moreover, the efficacy of therapy is hampered by the lack of available methods to quantify ROS and ROS damage in vivo in tissues and blood vessels, and a study design that considers patient differences in ROS-generating systems or cellular antioxidants [108,188]. It is also important to better understand the effects of oxidants and antioxidants in clinically relevant models of human disease [189]. Several alternative antioxidant-based experimental approaches are being developed, but further research and understanding of oxidative signaling is needed before they can be used in the treatment of CVD. Although there have been many animal studies with promising results, unfortunately, the results of randomized clinical trials do not support the positive effects of antioxidants on the cardiovascular system. Evidence from randomized clinical trials on the effectiveness of antioxidants in CVD, especially assessing their long-term effects, is still lacking.

Emerging therapies, however, represent a promising source for potential future clinical use. By regulating multiple genes involved in the response of biological systems to oxidative stress, miRNAs may represent biomarkers of CVD, including myocardial infarction, HF, or endothelial dysfunction. Modifying their expression may be a useful therapeutic option. In addition, the use of nanomaterials with unique ROS-regulating properties represents a promising option for the development of new therapeutic strategies for CVD.

## 6. Conclusions

ROS are small, highly reactive substances capable of oxidizing a wide range of molecules in the human body, including nucleic acids, proteins, lipids, carbohydrates, and even small inorganic compounds. The overproduction of ROS leads to oxidative stress, which constitutes a significant factor contributing to the development of diseases, especially cardiovascular.

CoQ10 inhibits the initiation of lipid peroxidation processes, thereby exerting a protective influence on the cardiac muscle and other tissues. Supplementation demonstrates favorable effects on cardiac function, correlating with a reduction in mortality and hospitalization rates in patients with HF. Moreover, it contributes to a reduction in inflammatory markers and total cholesterol levels concurrently increasing HDL-c concentrations. Evidence suggests that CoQ10 diminishes arterial blood pressure and mitigates vasoconstriction.

Effect of polyphenols on cardiovascular health are attributed to their direct antioxidant action, which involves scavenging ROS and reactive nitrogen species. Kaempferol might be used to lower the risk of atherosclerotic diseases by altering the expression of disease-related genes, such as MCP-1 or ICAM-1. It also inhibits ERK1/2 by downregulating cytokine receptors and reducing cardiac failure and hypertrophy. However, studies suggest that kaempferol may have mutagenic and genotoxic properties. Studies show that rutin improves cardiovascular structure and function, including hypertrophy, inflammation, and fibrosis. It prevents atherosclerosis by lowering total cholesterol, triglycerides, and insulin levels in rat blood. Resveratrol has been shown to improve in both systolic and diastolic function of the left ventricle as well as the left ventricular ejection fraction in patients with CHD.

Higher serum levels of carotenoids were correlated with a decreased risk of elevated serum NT-proBNP levels, which might indicate that carotenoids can partake in preventing cardiac overload. Moreover, they were associated with a reduced risk of atherosclerosis and inversely associated with inflammatory cytokines.

There is great hope that antioxidants and substances with antioxidant activity might minimize the negative effects of ROS and, at the same time, help to improve the prognosis of patients with CVD. Therefore, novel therapies based on antioxidant activity are researched, such as drugs targeting NRF2, which has a protective role against cardiomyocytes, XO inhibitors with a potential role in decreasing blood pressure, and NOX inhibitors with potent anti-atherosclerotic effects. In addition, more and more studies are emerging examining the potential impact of miRNAs on cardiovascular disease, but these remain in the animal phase. Similar is the case of nanoparticles, which have great potential but require further research.

Although there have been many animal studies with promising results, unfortunately, the results of randomized clinical trials do not support the positive effects of antioxidants on the cardiovascular system. Evidence from randomized clinical trials on the effectiveness of antioxidants in CVD, especially assessing their long-term effects, is still lacking. The effectiveness of antioxidant defenses is constrained by the role oxidative stress plays in disease pathology. Typically, oxidative stress is a secondary rather than a primary factor in disease, so mitigating it might not significantly alter disease progression. This limitation is frequently overlooked in clinical trials of antioxidants. Additionally, there is concern that compounds meant to boost antioxidant defenses may not achieve effective concentrations in vivo. Adopting a beneficial diet rich in antioxidant-containing foods over the long term is supported by evidence for reducing CVD risk factors. However, the same benefits are not conclusively seen with antioxidant supplements, whether these are short- or long-term clinical trials. The optimal timing for follow-up on the use of antioxidants to reduce cardiovascular disease risk factors remains uncertain and warrants further investigation due to the variability in outcomes observed across studies. For specific recommendations and duration, consulting with a healthcare provider is advisable.

This review has limitations. There was no specific selection criteria or research methodology used to identify sources and references for this review. In addition, due to the extent of the topic, the focus was on several major antioxidants and their potential role in the treatment of cardiovascular disease. The review has also strengths. We have presented overviews for the most known antioxidants, their chemical characteristics, and their impact on CVD diseases.

## Figures and Tables

**Table 2:** The role of different carotenoids in preventing CVD [97,111,113,120,121,122,123,125].

Carotenoids	Function	Role in Preventing CVD
β-Carotene	Inhibits LDL oxidation	Prevents atherosclerosis
	Decreases TNF-α-induced inflammation in endothelial cells	Reduces risk of CVD by opposing inflammatory oxidative stress
Lycopene	Decreases TNF-α-induced inflammation in endothelial cells	Reduces risk of CVD by opposing inflammatory oxidative stress
	Inhibits IL-1 secretion	Exerts an anti-atherogenic effect
	Increases NO levels	Dilates blood vessels, slowing the progression of atherosclerosis
	Regulates PCSK9 and HMGR genes, and increases LDL-R activity	Lowers hypercholesterolemia
	Improves the LDL/HDL ratio	Reduces the risk of atherosclerosis and postpones its progression
	Reduces accumulation of cholesterol in the aorta	
	Inhibits vascular smooth-muscle cell proliferation and foam cell formation	

**Table 3:** Role of miRNAs and the potential target of novel treatments for cardiovascular diseases. Abbreviations: ROS, reactive oxygen species; NOS, nitric oxide synthase; VEGF, vascular endothelial growth factor; CVD, cardiovascular disease.

Type of miRNA	Role in Onset of CVD
miRNA-210	During hypoxia, miRNA-210 promotes angiogenesis and inhibits cardiomyocyte apoptosis.
miRNA-1	Involved in the differentiation and proliferation of muscle cells. In anemic myocardium, it regulates cardiomyocyte growth and proapoptotic factors. Overexpression increases ROS production.
miRNA-133	Inhibition results in NOS production and may prevent endothelial dysfunction.
miRNA-92a	Regulates NOS expression, reduces plaque inflammation, and increases its stability by promoting cell proliferation and angiogenesis.
miRNA-206	Regulates VEGF expression, inhibits viability, and increases apoptosis of endothelial progenitor cells.
miRNA-377	Inhibition of miRNA-377 reduces myocardial fibrosis and improves its function.

**Table 4:** Types of nanoparticles and their functions related to prevention and treatment of cardiovascular diseases based on animal models.

Type of Nanoparticles	Function of Nanoparticles
H_2_O_2_-responsive nanoparticles	Anti-inflammatory and anti-apoptotic effects and reductions in further organ damage.
Nanoparticles with antioxidant properties	Enables imaging the thrombus and inhibits its formation by scavenging H_2_O_2_ and reduces oxidative stress.
Nanoparticles carrying SOD	Reduces myocyte apoptosis and improves myocardial function.
Nanoparticles carrying *N*-acetylcysteine	Attenuates myocardial fibrosis.
Selenium-based nanoparticles	Reduces ROS production in ischemic cardiomyocytes.

## Data Availability

The data used in this article were sourced from materials mentioned in the References Section.
